# Efficacy of grape seed hydro‐alcoholic extract in the treatment of *experimentally Pasteurella multocida* infected rabbits

**DOI:** 10.1002/vms3.446

**Published:** 2021-02-16

**Authors:** Sawsan M. A. El‐Sheikh, Fatma M. Youssef, Haidi I. Mohamed, Gaber El‐Saber Batiha, Ashraf Albrakati, Azza A. A. Galal

**Affiliations:** ^1^ Department of Pharmacology Faculty of Veterinary Medicine Zagazig University Zagazig Egypt; ^2^ Department of Clinical Pathology Animal Health Research Institute Ismailia Egypt; ^3^ Department of Pharmacology Animal Health Research Institute Ismailia Egypt; ^4^ Department of Pharmacology and Therapeutics Faculty of Veterinary Medicines Damanhour University Damanhour Egypt; ^5^ Department of Human Anatomy College of Medicine Taif University Taif Saudi Arabia

**Keywords:** GC‐MS, grape seed extract, *Pasteurella multocida*, rabbit

## Abstract

Pasteurellosis is one of the rabbit's most bacterial severe diseases and leads to considerable financial damages in large production systems worldwide. Antibiotic use in animals may lead to antibiotic residues in animal products, including meat. Therefore, this study was designed to evaluate the potential role of grape seed extract (GSE) in treating *Pasteurella multocida* infection in rabbits. For this purpose, 45 weaned male New Zealand rabbits were divided into three groups; control, infected and infected‐GSE treated. Experimental *P. multocida* infection in rabbits induced a remarkable decrease in body weight, body weight gain, as well as microcytic hypochromic anaemia, leucocytosis, neutrophilia and lymphocytopenia. Also, a significant increase in the hepatic and renal injury biomarkers, in interleukin‐6, total globulin, α, β and γ globulins, as well as a marked reduction in total protein and albumin, were recorded in the *P. multocida*‐infected rabbits. Treatment of infected rabbits with GSE modulated most of these altered parameters. This study endorses the administration of GSE for the treatment of Pasteurellosis in rabbits. Further studies are required to identify the possible additional effects, appropriate doses and duration of the GSE therapy in rabbits Pasteurellosis.

## Graphical abstract

According to the authors' knowledge, no comprehensive work was dedicated to assess the possible impact of grape seed extract in treating of *P. multocida* infection in rabbits. Moreover, grape seed extract could represent a promising for enhancement of Pasteurellosis treatment in rabbits and reduction of ofloxacin side effects.

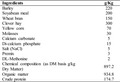

## 1 INTRODUCTION

Rabbit production is very important to improve the consumption of animal proteins in developing nations (Adeyinka, 2007). The occurrence of diseases is inevitable in any animal production unit and leads to prospective economic losses (Quesada et al., 2013). Pasteurellosis induced by *Pasteurella multocida* (*P. multocida)*, a virulent and readily transmitted coccobacillus, is one of the rabbit's most serious bacterial diseases and leads to great financial damages in big production systems worldwide (Takashima et al., 2001). This disease is characterized by multiple clinical symptoms, like respiratory distress, genital disorders, otitis, abscesses and septicaemia, but *P. multocida* infection may also asymptomatic (Jaglic et al., 2008). Rabbits could become infected with *P. multocida* instantly after birth, and the incidence of colonization rises with age to approximately 5 months. Most adult rabbits are thought to have been infected with *P. multocida* (Palócz et al., 2014).

Antibiotic use in animals may lead to antibiotic residues in animal products, including meat, milk and eggs. The most significant side effect of antibiotic residues is the transfer of antibiotic‐resistant bacteria to humans. It is critical to regulate the use of antibiotics in food‐producing animals to control the residues (Bacanli & Basaran, 2019). The inappropriate and uncontrolled use of antibiotics in the veterinary sector carries potential microbial resistance hazards that could affect not only the animal production unit but also human health and the environment (Quesada et al., 2013). Taking these facts into account, herbal medicine can give a wealth of attractive ways to fight drug resistance (Narayanan et al., 2011; Potroz & Cho, 2015). Natural ingredients exhibit a variety of biological activities (Abdellatief et al., 2017; El‐Sheikh et al., 2019) and can be used efficiently to manage diseases (Galal et al., 2016; Gupta & Birdi, 2017; Hassanin et al., 2020) and to ameliorate different toxicities (Elewa et al., 2019; El‐hady & Galal, 2018; Elsheikh et al., 2018; Galal et al., 2019; Mohamed et al., 2020; Osama et al., 2019).

Grape (*Vitis vinifera* L.) is one of the world's major fruit crops based on cultivated regions and economic value (Torregrosa et al., 2015). Grape seeds, a by‐product of the juice and wine industry, are a rich source of polyphenols (Lin et al., 2014). Grape seed extract (GSE) contains plant flavonoids, potent antioxidants and exert many health‐promoting effects (Kar et al., 2006). Resveratrol is a non‐flavonoid polyphenol found in grapes; its antioxidant capacity (Abdel‐Daim et al., 2019; Bungau et al., 2019; Yeung et al., 2019) exerted neuroprotection (Ibrahim et al., 2018) against several different oxidative insults. GSE has renoprotective (Albrahim & Robert, 2020), cardioprotective (Razmaraii et al., 2016) and hepatoprotective (Ali et al., 2015) effects. GSE can be used in broiler chicken diets as an effective natural antioxidant and immunostimulant (Hassan et al., 2016). Also, it has anti‐inflammatory effects via reduction in tumour necrosis factor‐α and interferon‐γ expression and down‐regulated NF‐κB signalling (Bibi et al., 2016).

Grape seed extract can interfere with the growth of a wide range of gram‐negative and Gram‐positive bacteria. It inhibits the growth of *Aeromonas hydrophila*, *Bacillus cereus*, *Enterococcus faecalis*, *Enterobacter aerogenes*, *Klebsiella pneumoniae*, *Escherichia coli*, *Mycobacterium smegmatis*, *Pseudomonas aeruginosa*, *Pseudomonas fluorescens*, *Salmonella enteritidis*, *Salmonella typhimurium*, *Proteus vulgaris*, *Staphylococcus aureus* and *Yersinia enterocolitica* (Baydar et al., 2004, 2006; Jayaprakasha et al., 2003; Özkan et al., 2004). It also has inhibitory effects against several viruses and fungi. GSE could be a potential source of antimicrobial agents in the food industry and the clinical environment (Memar et al., 2019).

The reduction of antibiotics use in the veterinary sector is of great importance. Therefore, this work was designed to assess the potential effect of GSE in treating *P. multocida* infection in rabbits. It has been achieved by assessing (a) antibacterial activity of GSE (b) haematological, immunological and biochemical parameters of *P. multocida*‐infected rabbits.

## 2 MATERIALS AND METHODS

### 2.1 Plant material and extraction

Grape seeds were purchased from a local market, Kasassen, Ismailia, Egypt. Grape seeds were dried in a hot air oven at 40ºC till complete dryness and pulverized to a fine powder in a mechanical grinder. The powder was soaked in 70% ethanol (25% w/v) for 72 hr in a dark place (25–30°C) and was stirred three times a day (Badavi et al., 2013). The extract was then filtered through Whatman No. 1 filter paper, and the residue was repeatedly extracted with the same solvent until it was colourless. The liquid extract was collected in a dark brown bottle. The extract was concentrated under reduced pressure in a rotary evaporator (temperature 40ºC) till complete dryness. The dried extract was collected, weighed and stored at 4°C until further use. The dried grape seed extract (GSE) extract was used for Gas Chromatography‐Mass Spectrometry (GC‐MS) analysis. Finally, the extract was reconstituted in 0.5% DMSO to be ready for administration.

### 2.2 Gas chromatography‐mass spectrometry analysis

The GC‐MS analysis of GSE was carried out at the National Research Center, El Dokki, Giza, Egypt. The GC‐MS analysis was performed using a TRACE GC Ultra Gas Chromatographs (THERMO Scientific Corp.), coupled with a THERMO mass spectrometer detector (ISQ Single Quadrupole Mass Spectrometer). TG‐MS fused silica capillary column (30 m × 0.25 mm × 0.1 µm film thickness).

### 2.3 *Pasteurella multocida* strain

*Pasteurella multocida* strain was obtained from the Animal Health Research Institute (AHRI), El Dokki Giza, Egypt. Colonies were suspended in sterile saline, and the density was adjusted with a final concentration of 1 × 10^7^ colony‐forming unit (CFU)/ml.

### 2.4 The antimicrobial activity of grape seed extract

The antibacterial activity of the GSE was determined by the disc diffusion method (Shrestha et al., 2012). Sterile paper discs (6 mm diameter) were loaded with 20 µl of each concentration of GSE (100, 200 and 500 µg/ml). Overnight cultures of *P. multocida* strain were diluted with sterile normal saline to give an inoculum size of 1 × 10^7^ CFU/ml. The inoculum was evenly spread on the surface of the dried nutrient agar plate by sterile spreader. The plates were incubated at 35°C for 30 min, then ofloxacin disc (5 µg, HiMedia Laboratories, Mumbai, India) and GSE discs were applied aseptically with gentle pressure. The plate was incubated at 37°C for 24 hr. The degree of sensitivity was determined by measuring the diameter of the inhibition zone around each disc (mm) with a graduated ruler.

### 2.5 Experimental rabbits and management

A total of 45 recently weaned male New Zealand rabbits, 1 month age, weighing about 600 ± 20 g were obtained from a rabbit farm in Kasaseen, Ismailia, Egypt. They were kept for one week before the commencement of the experiment. They were housed in a double flat galvanized wire, batteries (40 × 50 × 40 cm) and were kept under hygiene and environmental conditions. The rabbits were fed on commercial ration supplied from El‐Baraka Company, Kasaseen; Ismailia. The formulation and chemical composition of the basal diet are shown in Table 1. The rabbits were checked three times daily (at 6 a.m., 2 and 10 p.m.) for feed, water and mortality.

**TABLE 1:** Feed ingredients and chemical composition of the diet

Ingredients	g/Kg
Barley	220
Soyabean meal	200
Wheat bran	150
Clover hay	300
Yellow corn	70
Molasses	30
Calcium carbonate	5
Di‐calcium phosphate	15
Salt (NaCl)	5
Premix	3
DL‐Methonine	2
Chemical composition (as DM basis g/kg Dry Matter)	897.2
Organic matter	934.8
Crude protein	174.7

Each 3 kilograms of premix contains: Vit. A (12,000,000 IU); Vit. D3 (2,000,000 IU); Vit. E (10,000 mg); Vit K_3_ (2000 mg); Vit. B_6_ (1,500 mg); Vit.B_1_(1,000 mg); Vit B_12_ (10 mg); Vit.B_2_(5,000 mg); Biotin (50 mg); Coline Chloride (250,000 mg); Pantothenic acid (10,000 mg); Nicotinic acid (30,000 mg); Iron (30,000 mg); Folic acid (1,000 mg); Zinc (50,000 mg); Manganese (60,000 mg); Copper (10,000 mg): Iodine (1,000 mg); Selenium (100 mg); Cobalt (100 mg); CaCO_3_ (3,000 mg).

DE (Kcal/kg) = 4151 − (122*Ash) − (64*Fibre) + (38*Fat) + (23*CP)

#### 2.5.1 Induction of snuffle and experimental design

After 1 week of acclimatization, the rabbits (5 weeks of age) were randomly assigned into three equal groups (each of 15 rabbits). On 1st day of the experiment, group I (control group) intranasally instilled with 0.1 ml sterilized saline. In all other rabbits, snuffles were induced by intranasal instillation of 0.1 ml of *P. multocida* (1 × 10^7^ CFU) (Jarvinen et al., 1998).

Twenty‐four hours later (after the appearance of clinical signs), various treatments were started and continued for five consecutive days. Experimentally infected rabbits were allocated into group II (infected—non‐treated), were orally given 0.5% DMSO solution; group III (infected—GSE‐treated), was orally administered with GSE 250 mg/kg once daily (Benzer et al., 2012); The final volume of each treatment was adjusted to be 2 ml and administered using a plastic syringe connected to a rubber stomach tube. The rabbits in all groups were carefully observed throughout the study. Body weight (BW) was determined with an electronic balance on the 1st, 7th and 14th days of the experiment.

#### 2.5.2 Sampling

Two blood samples were collected from the ear vein from five rabbits of each group on the 1st and 7th day post‐treatment (i.e. 7th and 14th day of the experiment). Frist blood sample was collected in EDTA tube for haematological analysis. The second blood sample was collected in a clean, dry centrifuge tube and was centrifuged at 3,000 rpm for 10 min. The serum was separated and stored at −20°C for further investigations.

#### 2.5.3 Haematological analysis

Blood with EDTA used to determine erythrocytic (RBCs) count, packed cell volume (PCV), haemoglobin concentration (Hb), white blood cell (WBCs) count and the differential leucocytic count (Grindem, 2011).

#### 2.5.4 Biochemical analysis

Commercially available diagnostic kits (bioMérieux, France) were used for colorimetric determination of serum aspartate aminotransferase (AST), alanine aminotransferase (ALT) (Reitman & Frankel, 1957), total protein (Doumas et al., 1981), albumin level (Doumas et al., 1972), urea (Chevari & Ebst, 1975), creatinine (Larsen, 1972) and lactate dehydrogenase (LDH) (Buhl & Jackson, 1978) levels. The serum globulin was calculated by subtracting the albumin from the obtained total protein (Busher, 1990).

#### 2.5.5 Immunological studies

Serum protein electrophoresis was used for measuring different globulin fractions. It was done using sodium dodecyl sulphate polyacrylamide gel electrophoresis (SDS‐PAGE) (Smith, 1984) in the Biochemistry Department, Animal Health Research Institute, Ismailia, Egypt. Serum Interleukin‐6 **(**IL‐6) level was detected using a specific rabbit ELISA kit (MyBioSource) in following the manufacturer's procedures.

### 2.6 Statistical analysis

The obtained data were statistically analysed by a one‐way analysis of variance (ANOVA) method followed by Duncan multiple range tests to compare the means. Differences were considered significant at *p* < .05.

## 3 RESULTS

### 3.1 Chemical compositions of grape seed extract

**FIGURE 1:**
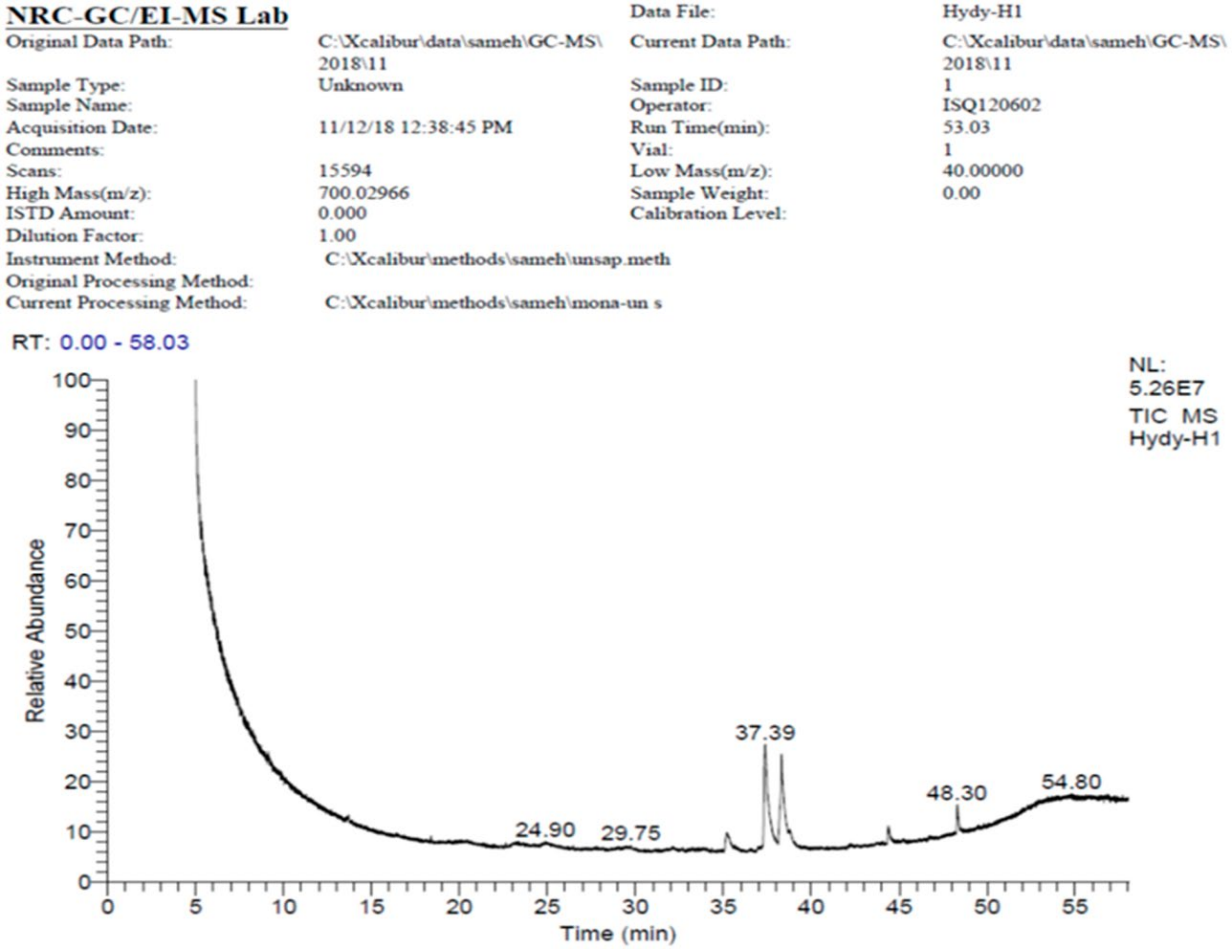
GC/MS chromatogram of grape seeds hydroalchoholic extract

The hydroalcoholic extraction of 1 kg of grape seed powder yield 28.64 g dry extract. The chemical compositions of GSE are illustrated in Table 2 & Figure 1. Overall, 79 compounds were clarified, constituting 97% of the total ingredients of GSE. Guanosine (20.55%), Glycerin (19.44%), Lycopresen (10.05%), 15‐Tetracosenoicacid, methyl ester (6.60%) and Octadecanoicacid, ethyl ester (6.14%) were the main compounds.

**TABLE 2:** Chemical composition of grape seeds hydroalchoholic extract analysed using GC–MS with relative percentages of components

Peak No.	Rt (min)	MF	MW	Area %	Identified compounds
1	6.14	C8H12	108	0.42	2Methylhept2,6dien4ol
2	6.29	C22H28N2O3	368	0.51	(1'Benzyl2'oxo1',2',3',4'tetrahydro4'pyridyl)2tertbutyl1aza3oxabicyclo[3.3.0]octan4one
3	6.49	C19H19N7O6	441	1.08	Folic Acid
4	6.66	C9H17Cl	160	0.59	6Chloro2,3,3trimethyl1hexene
5	6.94	C18H17NO	263	0.60	2,2dimethyl3,5d iphenyl2hpyrrole 1oxide
6	7.05	C27H36O8	488	0.99	(22S)21Acetoxy6à,11ádihydroxy16à,17àpropylmethylenedioxypregna1,4diene3,20dione
7	7.12	C15H24O4	268	0.55	Indeno[3a,4b]oxiren2ol,octahydro4amethyl5[(Tetrahydro2Hpyran2yl)oxy],
8	7.20	C10H20O3	188	0.71	(2R)2Methyl4[(tetra hydro2Hpyran2yl) oxy]butan1ol
9	7.26	C12H19N3O4	269	0.82	Pyrimidin2,4dione,1,2,3,4tetrahydro5methyl1[2hydroxymethyl3dimethylamino]tetrahydrofur5yl
10	7.36	C16H32N2	252	0.79	N(2Cyanoethyl)2methyldodecylamine
11	7.46	C7H12O	112	0.57	1Cyclobutyl cyclopropanol
12	7.61	C23H46O2	354	1.49	Docosanoic acid, methyl ester
13	7.67	C9H13NO2	167	0.51	N(3'Methylbut2' enoyl)2pyrrolidone
14	7.96	C11H15N5O3	265	0.30	LHistidinamide, 5oxoLprolyl
15	8.25	C20H36O2	308	0.60	2HPyran, tetrahydro2( 12penta decynyloxy)
16	8.73	C17H30O3	282	0.44	Tetrahydropyran Z10dodecenoate
17	8.80	C22H18	282	0.26	1(oxylyl) anthracene
18	9.13	C20H14N2	282	1.31	1,2Bis(2'quinolylmethyl)ethylene
19	9.33	C26H36O8	476	0.36	1HCyclopropa[3,4]benz[1,2e]azulene5,7b,9,9atetrol
20	9.47	C11H10BrFO2	272	0.49	3Methyl2butenoicacid,2bromo4fluorophenylester
21	9.79	C21H32O3	332	0.76	,5,6epoxy3hydroxy Pregnan20one
22	10.00	C15H18O3	246	0.38	10 (Tetrahydropyran2yloxy)tricyclo[4.2.1.1(2,5)]deca3,7dien9one
23	10.27	C9H14O	138	0.55	3methylenebicyclo[3.2.1]octan1ol
24	10.50	C26H54	366	0.26	3ethyl5(2ethylbutyl) Octadecane
25	11.16	C7H10ClNO	159	0.23	N,NDimethyl2Hpyran2iminiumchloride
26	11.54	C19H28O4	320	0.28	2Cyclohexene1carboxylicacid,2(7hydroxy3methyl1,3octaenyl)1,3dimethyl4oxo,methylester
27	11.85	C26H38N4O4	470	0.29	Ceanothine C
28	12.22	C22H41NO2	351	0.22	1[8(3octyloxiranyl)1oxooctyl Pyrrolidine
29	12.33	C18H17NO	263	0.28	2,2dimethyl 3,5dipheny l2hpyrrole 1oxide
30	12.48	C13H19N5O5	325	0.40	2one,4[Nmethylureido]1[4methylaminocarbonyloxymethyl Pyrimidin
31	12.89	C27H38O4S2	490	0.32	2á,4aEpoxymethylphenanthrene7methanol,1,1dimethyl2methoxy8(1,3dithiin2ylidene)methyl1,2,3,4,4a,4b,5,6,7,8,8a,9dodecahydro,acetate
32	13.18	C20H24O5	344	0.26	5amethyl3,8dimethylene2oxododecahydrooxireno[2',3':6,7]naphtho[1,2b]furan6yl2methyl2butenoate
33	13.26	C26H34O11	522	0.29	4HCyclopropa[5',6']benz[1',2':7,8]azuleno[5,6b]oxiren4one,8,8abis(acetyloxy)2a[(acetyloxy)methyl]1,1a,1b,1c,2a,3,3a,6a,6b,7,8,8adodecahydro3,3a,6btrihydroxy1,1,5,7tetramethy
34	13.53	C21H25NO2S	355	0.23	trans2Cyclohexyl3(2methylphenyl)4phenyl1,2thiazetizine1,1dioxide
35	13.68	C22H17N3O2	355	1.17	3(Maleimido2'yl)1methyl2(1'methylindol2'yl)indole
36	16.42	C21H28O3	328	0.65	4,6Androstadien3áol17one,acetate
37	16.55	C20H28O6	364	1.06	Effusanin E
38	18.37	C26H20Cl2N2	430	0.74	1,3Bis(4chlorobenzyl)5,6dihydrobenzoquinazoline
39	20.40	C42H64O2	600	0.42	,1,1',2,2'tetrahydro1,1 dimethoxy.Carotene
40	20.61	C14H23N3O10	393	0.24	Pentetic Acid
41	23.02	C24H32O8	448	0.32	5HCyclopropa[3,4]benz[1,2e]azulen5one,9(acetyloxy)3[(acetyloxy)methyl]1,1a,1b,4,4a,7a,7b,8,9,9adecahydro4a,7b,9atrihydroxy1,1,6,8tetramethyl
42	23.07	C27H38O8	490	0.28	2Butenoicacid,2methyl,2(acetyloxy)1,1a,2,3,4,6,7,10,11,11adecahydro7,10dihydroxy1,1,3,6,9pentamethyl4a,7aepoxy5Hcyclopenta[a]cyclopropa[f]cycloundecen11ylester7a
43	23.14	C12H20O	180	0.33	(+)5cyclohexyl6hexenal
44	24.90	C26H44O5	436	1.60	Ethyl isoallocholate
45	28.17	C38H76O2	564	0.33	Octadecanoicacid,eicosyl ester
46	29.05	C15H20O5	280	0.24	Tetraneurin Adiol
47	29.75	C22H23NO8	429	0.47	(6 O methylated flavonols)
48	32.68	C18H16O7	344	0.58	7,3',4'trimethoxy Quercetin
49	33.25	C35H48O3S	548	0.48	9(11)Dehydroergosteroltosylate
50	33.85	C36H58O6	586	0.36	Hexadecanoicacid,1a,2,5,5a,6,9,10,10aoctahydro5,5adihydroxy4(hydroxymethyl)1,1,7,9tetramethyl11oxo1H2,8amethanocyclopenta[a]cyclopropa[e]cyclodecen6ylester
51	35.21	C20H40O2	312	6.14	Octadecanoicacid,ethyl ester
52	35.65	C21H34O2	318	0.39	17one,3ethyl3hydroxy Androstan
53	36.58	C22H40O7	416	0.32	Agaricic acid
54	36.97	C29H28N2O6S	532	0.42	4[3(3,4Dimethoxyphenyl)1oxo11thiophenyl1,2,3,4,5,11hexahydrodibenzo[B,E][1,4]Diazepin10yl]4oxobuteric acid
55	37.11	C3H8O3	82	19.44	Glycerin
56	37.37	C14H16	184	2.97	Pentacyclo[7.2.1.0(2,7).0(2,8).0(3,8)]dodec10en12spiro1'cyclopropane
57	38.32	C25H48O2	380	6.60	15‐Tetracosenoicacid, methyl ester
58	42.12	C21H26N2O2	338	0.32	1acetyl20àhydroxy16methylene Strychane,
59	42.20	C26H50	362	3.62	1,1'dodecylidenebis[4methyl Cyclohexane,
60	42.27	C42H64O2	600	0.51	.,1,1',2,2'tetrahydro1,1'dimethoxy Carotene
61	43.70	C35H46O8	594	0.40	2,4,6Decatrienoicacid,1a,2,5,5a,6,9,10,10aoctahydro5,5adihydroxy4(hydroxymethyl)1,7,9trimethyl1[[(2methyl1oxo2butenyl)oxymethyl]11oxo1H2,8amethanocyclopenta[a]cyclopropa[e]cyclodecen6ylester
62	43.85	C10H13N5O5	283	20.55	Guanosine
63	44.10	C16H32O2	256	0.89	Palmitic acid
64	44.38	C22H23N	301	3.42	2,6dimethylN(2methylàphenylbenzyl)aniline
65	44.99	C32H54O4	502	0.25	7,8Epoxylanostan11ol,3acetoxy
66	46.15	C35H68O5	568	0.39	Hexadecanoic acid,1(hydroxymethyl)1,2ethanediyl ester
67	46.70	C26H34O11	522	0.59	4HCyclopropa[5',6']benz[1',2':7,8]azuleno[5,6b]oxiren4one,8,8abis(acetyloxy)2a[(acetyloxy)methyl]1,1a,1b,1c,2a,3,3a,6a,6b,7,8,8adodecahydro3,3a,6btrihydroxy1,1,5,7tetramethyl
68	48.30	C40H66	546	10.05	Lycopersen
69	49.50	C22H36N6O2	416	0.31	1,2Bis[1(2hydroxyethyl)3,6diazahomoadamantantydene9]hydrazine
70	49.81	C22H25NO7	415	0.28	IsocholchiIfoline

Abbreviations: Rt, Retention time; MF, Molecular formula; MW, Molecular weight.

### 3.2 Antibacterial activity

*P. multocida* showed various degrees of sensitivity to different GSE concentrations. Inhibition zones were 12, 15 and 20 mm for 100, 200 and 500 µg GSE/ml respectively. There was a positive correlation between GSE concentrations and inhibition zones. The highest inhibition was obtained by ofloxacin (35 mm).

### 3.3 Clinical symptoms, mortality rate and body weight changes in rabbits in response to *P. multocida* infection and oral administration of GSE

On the second day of the experiment, group II rabbits (infected—non‐treated) exhibited inconsistent clinical signs. These signs were mostly depression, off food, sneezing, nasal discharges, rough fur, laboured breathing, and in few cases, there were subcutaneous abscesses and also diarrhoea. However, rabbits treated GSE (group III) revealed less severe clinical signs than did group II. During the experimental period, five rabbits in the infected non‐treated group, three rabbits in the infected GSE‐treated group died.

Experimental *P. multocida* infection induced a significant (*p* < .05) decrease in the BW and body weight gain (BWG) of the infected group in contrast to the control group on the 7th and 14th day of the experiment. Meanwhile, GSE‐treated group showed a significant (*p* < .05) increase in the BW and BWG in contrast to the infected group on the 7th and 14th day of the experiment, as shown in Table 3.

**TABLE 3:** Body weight changes of rabbits in response to *P. multocida* infection and oral administration of GSE

Parameters	Groups	Experimental days		
		1st	7th	14th
Body weight (g)	I	748.8 ± 10.55	875.2 ± 9.6^a^	999.2 ± 8.45^a^
	II	729.8 ± 2.9	706.8 ± 3.45^c^	786.8 ± 3.9^c^
	III	733.8 ± 9.6	840.2 ± 12^b^	947.4 ± 14^b^
Weight gain (g)	I	N/A	126.4 ± 1.7^a^	124 ± 1.7^a^
	II	N/A	−23 ± 2^c^	80 ± 3.53^c^
	III	N/A	106.4 ± 5.6^b^	107.2 ± 4.35^b^

Note

Values are represented as the mean ± SE (*n* = 5). Group I (control group) intranasally administered with 0.1 ml sterilized saline; group II (infected—non treated), infected group were orally administered with 0.5% DMSO solution; group III (infected—GSE‐treated), infected rabbits were orally administered with GSE 250 mg/kg once daily. Column carrying different superscripts (a, b, c) are significantly different at *p* < .05.

### 3.4 Effects of oral administration of GSE on haematological parameters in *P. multocida* experimentally infected rabbits

On the 1st day post‐treatment, intranasal inoculation of rabbits with *P. multocida* caused a significant (*p* < .05) reduction in RBC count, PCV, Hb concentration, MCH, MCHC, platelets and lymphocytes and a significant (*p* < .05) elevation in MCV, total WBC count, neutrophil, monocytes and eosinophils in contrast to the control group. While oral administration of infected rabbits with GSE for 5 days significantly (*p* < .05) elevated RBC count, PCV, Hb concentration, MCH, MCHC, platelets and lymphocytes and significantly reduced total leucocyte count, eosinophils and monocytes in contrast to the non‐treated‐infected group.

On the 7th day post‐treatment, a significant (*p* < .05) reduction in RBC count, Hb concentration, PCV, MCH, MCHC, MCV, platelets and neutrophils and a significant (*p* < .05) elevation in total WBC count, lymphocytes, monocytes and eosinophils was recorded in infected non‐treated group in contrast to the control group. While oral administration of infected rabbits with GSE for 5 days significantly (*p* < .05) elevation RBC count, Hb concentration, PCV, MCH, MCHC, platelets and neutrophils and significantly decreased total leucocyte count, lymphocytes, eosinophils and monocytes in contrast to the non‐treated‐infected group (Table 4).

**TABLE 4:** Effects of oral administration of GSE on haematological parameters in *P‐multocida* experimentally infected rabbits

Days post treatment	Parameters	Groups		
		I	II	III
1st day	RBCs (10^6^/µl)	3.25 ± 0.15^a^	2.45 ± 0.22^c^	2.6 ± 0.17^b^
	Hb (g/dl)	11.1 ± 0.40^a^	6.65 ± 0.30^c^	8.07 ± 0.41^b^
	PCV (%)	23.84 ± 1.17^a^	19.43 ± 0.001^c^	20.43 ± 0.05^b^
	MCV(fl)	73.35 ± 0.41^b^	79.31 ± 0.83^a^	78.58 ± 2.54^a^
	MCH (pg)	34.15 ± 0.39^a^	27.14 ± 0.61^c^	31.04 ± 1.26^b^
	MCHC (%)	46.56 ± 0.39^a^	34.23 ± 0.61^c^	39.5 ± 0.6^b^
	Platelets	182 ± 10^a^	94.5 ± 11.6^c^	124.33 ± 12.5^b^
	WBCs (10^3^/µl)	4.35 ± 0.85^c^	8.7 ± 0.01^a^	7.53 ± 1.72^b^
	Heterophils (%)	41.67 ± 1.7^b^	50.00 ± 2.1^a^	46.00 ± 1.5^a^
	Lymphocytes (%)	50.33 ± 2.3^a^	36.00 ± 1.0^c^	43.00 ± 1.5^b^
	Monocytes (%)	5.00 ± 0.31^c^	10.00 ± 0.27^a^	7.21 ± 0.22^b^
	Eosinophils (%)	3.00 ± 0.2^c^	4.00 ± 0.3^a^	3.79 ± 0.4^b^
7th day	RBCs (10^6^/µl)	2.96 ± 0.3^a^	2.56 ± 0.15^c^	2.8 ± 0.14^b^
	Hb (g/dl)	9.96 ± 0.44^a^	7.24 ± 0.79^c^	8.28 ± 0.53^b^
	PCV (%)	22.84 ± 1.98^a^	17.84 ± 1.1^c^	20.13 ± 1.36^b^
	MCV (fl)	77.16 ± 2.84^a^	67.32 ± 1.92^b^	71.89 ± 2.30^b^
	MCH (pg)	33.65 ± 1.9^a^	28.28 ± 0.89^c^	29.57 ± 1.5^b^
	MCHC (%)	43.61 ± 1.9^a^	40.58 ± 0.89^c^	41.13 ± 1.78^b^
	Platelets	152.60 ± 2.75^a^	121.8 ± 3.87^c^	130 ± 3.70^b^
	WBCs (10^3^/µl)	9.62 ± 0.41^b^	12.1 ± 0.23^a^	9.98 ± 0.70^b^
	Heterophils (%)	43 ± 0.5^a^	26 ± 0.61^c^	36 ± 0.48^b^
	Lymphocytes (%)	50 ± 1.2^c^	60 ± 1.5^a^	52 ± 1.35^b^
	Monocytes (%)	4 ± 0.1^c^	8 ± 0.3^a^	7 ± 0.70^b^
	Eosinophils (%)	3 ± 0.3^c^	6 ± 0.4^a^	5 ± 0.4^b^

Note

Values are represented as the mean ± SE (*n* = 5). Group I (control group) intranasally administered with 0.1 ml sterilized saline; group II (infected—non‐treated), infected group were orally administered with 0.5% DMSO solution; group III (infected—GSE‐treated), infected rabbits were orally administered with GSE 250 mg/kg once daily. Row carrying different superscripts (a, b, c) are significantly different at *p* < .05.

Abbreviations: RBCs, Red blood cells; PCV, Packed cell volume; Hb, Haemoglobin; MCV, Mean corpuscular volume; MCHC, Mean corpuscular haemoglobin concentration; WBCs, White blood cells.

### 3.5 Effects of oral administration of GSE on biochemical parameters in *P. multocida* experimentally infected rabbits

On the 1st and 7th day post‐treatment, a significant (*p* < .05) elevation in the levels of serum ALT, AST, LDH, urea, creatinine and IL‐6 were reported in the infected non‐treated group in contrast to the control group. Meanwhile, oral administration of infected rabbits with GSE for 5 days significantly (*p* < .05) reduced the levels of serum ALT, AST, LDH, urea, creatinine and IL‐6 in contrast to the infected group (Table 4).

On the 1st day post‐treatment, experimental *P‐ multocida* infection in rabbits induced a significant (*p* < .05) reduction in the levels of serum total protein and albumin and a significant (*p* < .05) elevation in total globulin, α and β globulins in the infected non‐treated group. Meanwhile, oral administration of infected rabbits with GSE for 5 days significantly (*p* < .05) elevated the albumin levels in contrast to infected non‐treated group.

On the 7th day post‐treatment, a significant (*p* < .05) reduction in the levels of serum total protein and albumin and a significant (*p* < .05) elevation in total globulin, α and γ globulins was recorded in the infected non‐treated group in contrast to the control group. Meanwhile, oral administration of infected rabbits with GSE for 5 days significantly (*p* < .05) elevated the levels of serum total protein, albumin, total globulin, α and γ globulins in contrast to the infected non‐treated group (Table 5).

**TABLE 5:** Effects of oral administration of GSE on biochemical parameters in *P‐multocida* experimentally infected rabbits

Days post treatment	Parameters	Groups		
		I	II	III
1st day	AST (U/L)	62.9 ± 2.14^c^	144.46 ± 5.60^a^	137.68 ± 7.06^b^
	ALT (U/L)	30.02 ± 3.55^c^	45.6 ± 3.20^a^	43.50 ± 4.73^b^
	Urea (mg/dl)	29.42 ± 1.09^c^	72.74 ± 6.22^a^	46.08 ± 2.26^b^
	Creatinine (mg/dl)	0.63 ± 0.05^c^	1.01 ± 0.02^a^	0.95 ± 0.03^b^
	LDH (U/L)	148 ± 10.43^c^	602.32 ± 24.49^a^	509.16 ± 27.14^b^
	Total protein (g/dl)	8 ± 0.12^a^	6.89 ± 0.17^b^	7.09 ± 0.14^b^
	Albumin (g/dl)	5.88 ± 0.17^a^	2.77 ± 0.14^c^	3.04 ± 0.24^b^
	Globulin (g/dl)	2.12 ± 0.20 ^b^	4.12 ± 0.34^a^	4.05 ± 0.20 ^a^
	α globulin (g/dl)	0.72 ± 0.04^b^	1.79 ± 0.03^a^	1.62 ± 0.04^a^
	Β globulin (g/dl)	0.69 ± 0.03^b^	1.63 ± 0.11^a^	1.66 ± 0.07^a^
	γ globulin (g/dl)	0.71 ± 0.04^a^	0.7 ± 0.05^a^	0.77 ± 0.03^a^
	IL−6 (pg/ml)	0.29 ± 0.03^c^	2.13 ± 0.22^a^	1.21 ± 0.11^b^
7th day	AST (U/L)	53.06 ± 4.12^c^	92.16 ± 3.36^a^	74.62 ± 4.48^b^
	ALT (U/L)	28.63 ± 0.43^c^	42.46 ± 0.82^a^	38.35 ± 0.89^b^
	Urea (mg/dl)	35.74 ± 1.54^c^	44.04 ± 0.30^a^	40.48 ± 1.46 ^b^
	Creatinine (mg/dl)	0.70 ± 0.03^c^	0.82 ± 0.03^a^	0.78 ± 0.02^b^
	LDH (U/L)	126.63 ± 6.38^e^	421.57 ± 7.04^a^	205.25 ± 9.90^b^
	Total protein(g/dl)	8.21 ± 0.24^a^	4.43 ± 0.33^c^	7.54 ± 0.36^b^
	Albumin (g/dl)	6.40 ± 0.38^a^	2.11 ± 0.13^c^	3.95 ± 0.26^c^
	Globulin (g/dl)	1.81 ± 0.12^c^	2.32 ± 0.12^b^	3.59 ± 0.33^b^
	α globulin (g/dl)	0.38 ± 0.09^c^	1.44 ± 0.07^b^	1.62 ± 0.05^a^
	Β globulin (g/dl)	0.72 ± 0.04^a^	0.68 ± 0.05^a^	0.67 ± 0.02^a^
	γ globulin (g/dl)	0.71 ± 0.04^c^	1.20 ± 0.02^b^	1.30 ± 0.07^a^
	IL−6 (pg/ml)	0.36 ± 0.03^c^	2.14 ± 0.05^a^	1.65 ± 0.04^b^

Note

Values are represented as the mean ± SE (*n* = 5). Group I (control group) intranasally administered with 0.1 ml sterilized saline; group II (infected—non‐treated), infected group were orally administered with 0.5% DMSO solution; group III (infected—GSE‐treated), infected rabbits were orally administered with GSE 250 mg/kg once daily. Row carrying different superscripts (a, b, c) are significantly different at *p* < .05.

Abbreviations: ALT, Alanine Aminotransferase; AST, Aspartate transaminase; LDH, Lactate dehydrogenase; IL‐6, Interleukin‐6.

## 4 DISCUSSION

Pasteurellosis induced by *P. multocida*, a virulent and readily transmitted coccobacillus, is one of the rabbit's most serious bacterial diseases and leads to significant financial damages in big production systems worldwide (Takashima et al., 2001). The reduction of antibiotics uses in the veterinary sector attracts researchers for seeking natural antibacterial agents. Therefore, this work was designed to assess the potential role of GSEin treating *P. multocida* infection in rabbits.

*P. multocida* showed various degrees of sensitivity to different GSE concentrations. There was a positive correlation between GSE concentrations and inhibition zones. The antimicrobial effects of GSE are due to high levels of potent antioxidant polyphenols. The antibacterial effect of GSE, was bactericidal that was obtained by a disruption of the bacterial cell wall (Al‐Habib et al., 2010). These results were confirmed by the studies of (Filocamo et al., 2015; Shrestha et al., 2012). Sneezing, nasal discharges, laboured breathing are the most common clinical symptoms caused by *P. multocida* infection in rabbits, which is also the main cause of mortality and morbidity. These findings were supported by histopathological findings of the lung that recorded in our previously published paper. In a few cases, subcutaneous abscesses and diarrhoea were recorded in the infected group. The infected group revealed a marked reduction in BW and BWG all over the study period in contrast to the control group. This is due to off food and poor appetite. These results were supported by the studies (Alam et al., 2018; Edrees et al., 2017). Infected groups treated with GSE for five successive days showed milder clinical symptoms, reduction in mortality rate, and significant improvement of BW and BWG compared to the infected non‐treated group. These results may be attributed to the antibacterial effect of GSE. The phytogenic additives from plant extracts have been documented to promote higher digestibility of the nutrient, elevate the activity of the digestive enzyme and the secretion of gastric and pancreatic juice, protect the intestinal microvilli and enhance bird performance via antimicrobial effect (Hernandez et al., 2004).

In this study, intranasal inoculation of rabbits with *P. multocida* caused a marked reduction in RBCs count, PCV% and Hb concentration as well as macrocytic hypochromic anaemia (on 1st day post‐treatment) and microcytic hypochromic anaemia (on 7th day post‐treatment) in contrast to the control group. Reduction of RBCs count may be due to the impact of *P. multocida* endotoxins on RBCs, which leads to a reduction in their life span. Macrocytic hypochromic anaemia, probably due to activated erythropoiesis as a reaction of the bone marrow to the blood loss in the trachea‐pulmonary haemorrhage, induced by septicaemia (Feldman et al., 2000). Meanwhile, the most common cause of microcytic hypochromic anaemia observed during infection is decreased iron reserves of the body (Massey, 1992), which may be due to a massive increase in IL‐6, which promotes the production and release of hepcidin from the liver, thereby decreasing the iron carrier protein ferroportin, so the access of iron to the circulation is limited (Nemeth et al., 2004). Moving iron to storage in the mononuclear phagocytic system and using iron by bacteria renders it less accessible as an erythroid precursor (Walton, 2013).

The innate response is the first line of defence that occurs immediately in which neutrophils, macrophages, monocytes and eosinophils are involved (Carrillo et al., 2017). *P. multocida* experimental infection in rabbits induced leucocytosis, neutrophilia, monocytosis, eosinophilia and lymphocytopenia. Leucocytes were elevated to overcome infection as the normal reaction of bone marrow to infection leads to an increase in the number of WBCs predominantly polymorphonuclear leucocytes (neutrophil and eosinophils) (Abramson & Melton, 2000). Lymphocytopenia is commonly accompanied by a rise in neutrophil count in various infectious causes (Hawkins et al., 2006; Seebach et al., 1997; Wyllie et al., 2004). Our haematological results were supported by those of (Alam et al., 2018; Edrees et al., 2017). The treatment of *P. multocida*‐infected rabbits with GSE improves the haematological status compared to infected non‐treated rabbits. Cytokines are multifunctional mediators of the adaptive and innate immune system, stimulating and regulating inflammatory processes, playing a key role in the large network of interacting cells and signalling immune response‐related molecules (Eder et al., 2008). IL‐6 possesses pro‐ and anti‐inflammatory activities, and it has a broad effect on the immune system cells (Hunter & Jones, 2015). Our results revealed that *P. multocida* experimental infection in rabbits induced significant elevation of serum IL‐6. The IL‐6 elevation after the infection is an inflammatory reaction to control neutrophil and monocyte transition during the inflammatory process (Kaplanski et al., 2003). The treatment of *P. multocida‐*infected rabbits with GSE significantly reduced serum IL‐6 levels compared to infected non treated rabbits. This results due to the anti‐inflammatory effect of GSE (Bibi et al., 2016; Silvan et al., 2017; Yang et al., 2014).

Hypoprotenaemia, hypoalbuminaemia and hyperglobulinaemia were noted in *P. multocida*‐infected group all over the experimental period in contrast to the control group, which may be attributed to off food, poor appetite and the inability of the liver to synthesize proteins. Hyperglobulinaemia may be one of the immune system responses against *P. multocida* infection. Meanwhile, oral administration of infected rabbits with GSE for five days significantly (*p* < .05) increased the levels of serum total protein, albumin, total globulin, α and γ globulins in contrast to the infected non‐treated group. These results may be attributed to improving the appetite of rabbits, reduction in the damaging effects of bacteria on the liver, and immunomodulating outcomes of the testing agent.

A marked elevation in the serum levels of ALT, AST, LDH, urea and creatinine was recorded in *P. multocida*‐infected group all over the experimental period compared with the control group, which could be due to the worse effect of micro‐organism or its toxin on the liver and kidney. However, infected rabbits treated with GSE revealed a significant decrease in serum ALT, AST, LDH, urea and creatinine levels in contrast to the infected‐non‐treated group. These findings were supported by histopathological findings of liver and kidney that recorded in our previously published paper (El‐Sheikh et al., 2020). These results confirmed the antibacterial activity, hepato‐renal protective effect of GSE that reduced the harmful effects of bacteria on the liver and kidney. These results may be attributed to the antioxidant activity of GSE or its active constituents (Abdel‐Daim et al., 2019; Albrahim & Robert, 2020; Ali et al., 2015). The hepato‐renal protective effect and antibacterial activity of GSE were supported by those of (Hasona et al., 2017; Khalil, 2018).

## 5 CONCLUSION

*Pasteurella multocida* infection remains a major public health problem in rabbits. It leads to high economic loss due to disturbance in all body parameters, including growth performance, haematological, biochemical and immunological parameters. Our study demonstrated that GSE has a potential therapeutic role in treating Pasteurellosis in rabbits. This study endorses the administration of GSE as a useful therapeutic tool demonstrating the potential for enhancement of Pasteurellosis treatment in rabbits. Further studies are required to identify the possible additional effects, appropriate doses and duration of the GSE therapy in rabbits Pasteurellosis.

## CONFLICT OF INTEREST

The authors declare that they have no conflict of interests.

## AUTHOR CONTRIBUTION

Sawsan El‐Sheikh: Conceptualization; Data curation; Formal analysis; Supervision. Fatma M. Youssef: Investigation; Methodology. Haidi I. Mohamed: Investigation; Methodology; Validation. Gaber Batiha: Visualization; Writing‐original draft. Ashraf Albrakati: Funding acquisition; Resources. Azza Galal: Resources; Software; Writing‐review & editing.

### PEER REVIEW

The peer review history for this article is available at https://publons.com/publon/10.1002/vms3.446.
